# Integrating Palliative Care in the Intensive Care Unit for a Lethal Cervical Arteriovenous Malformation in a Reproductive-Aged Woman: A Case Report

**DOI:** 10.7759/cureus.99691

**Published:** 2025-12-20

**Authors:** Valentia Dannucio, Mario Madruga, Steve Carlan

**Affiliations:** 1 Internal Medicine, Orlando Regional Medical Center, Orlando, USA; 2 Academic Affairs and Research, Orlando Regional Medical Center, Orlando, USA

**Keywords:** ateriovenous malformation, end-of-life support, medical intensive care unit (micu), spontaneous hemorrhage, supportive and palliative care

## Abstract

Arteriovenous malformations (AVMs) of the head and neck are rare congenital vascular anomalies. Despite advances in endovascular techniques, extensive lesions can be difficult to manage and may lead to high morbidity. In rare cases, AVMs can become life-threatening, especially when located in the neck, where airway and vascular involvement can cause uncontrollable hemorrhage. Although intensive and palliative care are often seen as opposing approaches, they share common goals of symptom management, communication, and patient-centered decision-making in life-threatening illnesses.

We describe the case of a 38-year-old woman with a large, diffuse neck AVM located in the left parotid gland, extending into the left masticator space and downward. The primary arterial supply was from the left external carotid artery. The AVM was complicated by recurrent bleeding and airway obstruction. Despite multiple treatments, including endovascular embolization, vincristine therapy, and surgery, the patient experienced persistent bleeding, repeated shock episodes, and a gradual decline leading to death during a 36-day hospital stay. Palliative care was brought in on hospital day 8 for symptom control, anxiety management, and family support.

This case demonstrates the challenges of managing an aggressive vascular lesion and underscores the critical importance of early, concurrent integration of palliative care within the ICU paradigm for patients with complex, life-limiting illnesses. Early palliative involvement allowed for proactive symptom control, clarified care goals, and offered psychosocial support to her family. Ultimately, after compassionate withdrawal of life-sustaining treatments and organ donation, the patient passed away. This case emphasizes the significance of early, structured palliative care in ICU settings, particularly in conditions with poor prognosis despite maximum intervention.

## Introduction

Arteriovenous malformations (AVMs) of the head and neck are rare congenital vascular anomalies, accounting for about 1.5% of all vascular malformations, with up to 50% occurring in the oral and maxillofacial region [1,2]. These high-flow lesions feature direct arterial-venous connections without an intervening capillary bed, leading to progressive vascular expansion, tissue infiltration, and a risk of catastrophic hemorrhage.

Diagnosis relies on clinical presentation, imaging with computed tomography (CT) and magnetic resonance (MR) angiography [3], and confirmatory digital subtraction angiography [4]. Management generally involves multimodal therapy, including embolization, surgery, and adjunctive medical treatments, such as sirolimus or vincristine, administered by multidisciplinary vascular anomalies teams [5]. Despite advances in endovascular techniques, diffuse lesions often remain challenging to control, with high recurrence rates and significant morbidity [3]. In rare cases, these AVMs become lethal, especially when located in the neck, where airway and vascular involvement can cause uncontrollable hemorrhage. When curative or stabilizing options are exhausted, palliative care, defined by the WHO as “an approach that improves the quality of life of patients and their families facing life-threatening illness, through prevention and relief of suffering,” may be appropriate. Establishing a definitive diagnosis for complex vascular anomalies can be challenging, a difficulty previously reported in rare head and neck vascular lesions [1-4], where delayed or complex diagnostic pathways have been associated with fatal outcomes, underscoring the importance of early integration of palliative care when prognosis remains poor despite maximal intervention. However, integrating palliative care into the ICU, which traditionally focuses on recovery, can be difficult due to differing expectations, limited communication training, and misconceptions that palliative care means “giving up” [6].

We present a case of a 38-year-old woman with a diffuse cervical AVM who experienced recurrent life-threatening bleeding, illustrating both the natural progression of this disease and the evolving role of palliative care in the ICU.

## Case presentation

A 38-year-old woman with a history of a large cervical AVM (status post two embolizations) presented to the emergency department (ED) with two days of worsening dental bleeding, pain, and hemoptysis. She denied smoking, alcohol, or illicit drug use. She did not use aspirin. Her obstetric history included two prior term pregnancies delivered vaginally without complications. Her home medications included tranexamic acid (TXA) mouth rinse and iron supplementation.

**Figure 1:**
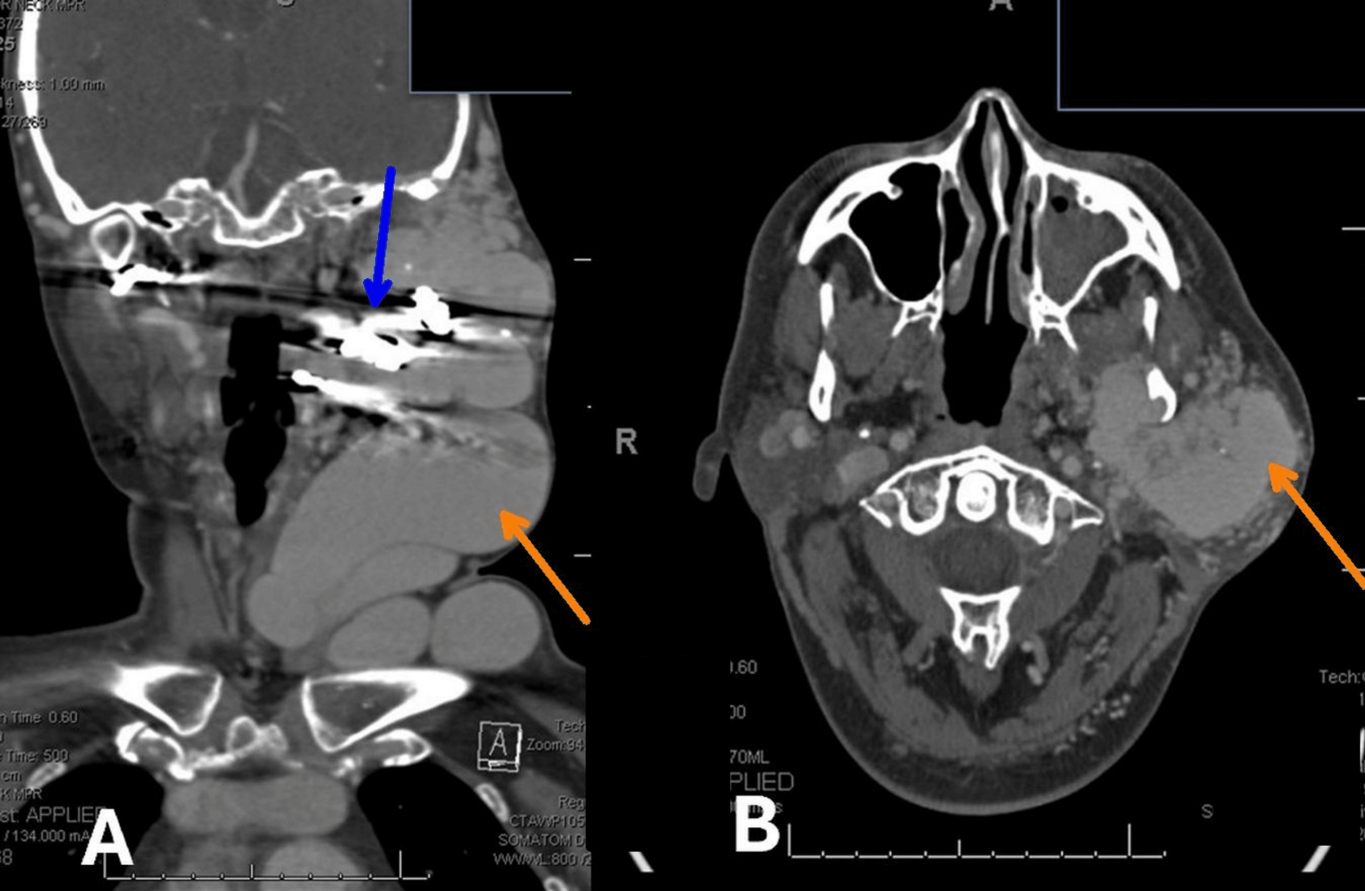
Contrast-enhanced CT angiography of the head and neck. Image A is a lateral view and shows some contrast with stasis from blockage going toward the large mass, which is getting minimal, if any, flow post-embolization. The orange arrow indicates the region of prominent abnormal vascular channels corresponding to the AVM nidus. The lesion is centered in the left parotid gland, with extension into the left masticator space and inferiorly into the upper neck. The blue arrow indicates the main arterial feeder from the left external carotid artery. Image B is an axial view with orange arrow at large mass with a few specks of white residual blood left after embolization. Yellow area shows residual large mass involving cheek, tongue, or throat, and neck. There appear to be other lobular masses lower with no vascularity that may have arteries elsewhere.

One month prior to presenting at the ED, she had an outpatient contrast-enhanced CT angiography of the head and neck, demonstrating a persistent large enhancing serpentine vascular malformation involving the left lateral face and neck, consistent with an AVM. The lesion was centered in the left parotid gland, with extension into the left masticator space and inferiorly into the upper neck. Arterial feeders were supplied from branches of the bilateral carotid arteries, predominantly from the left external carotid artery (Figure 1).

**Figure 2:**
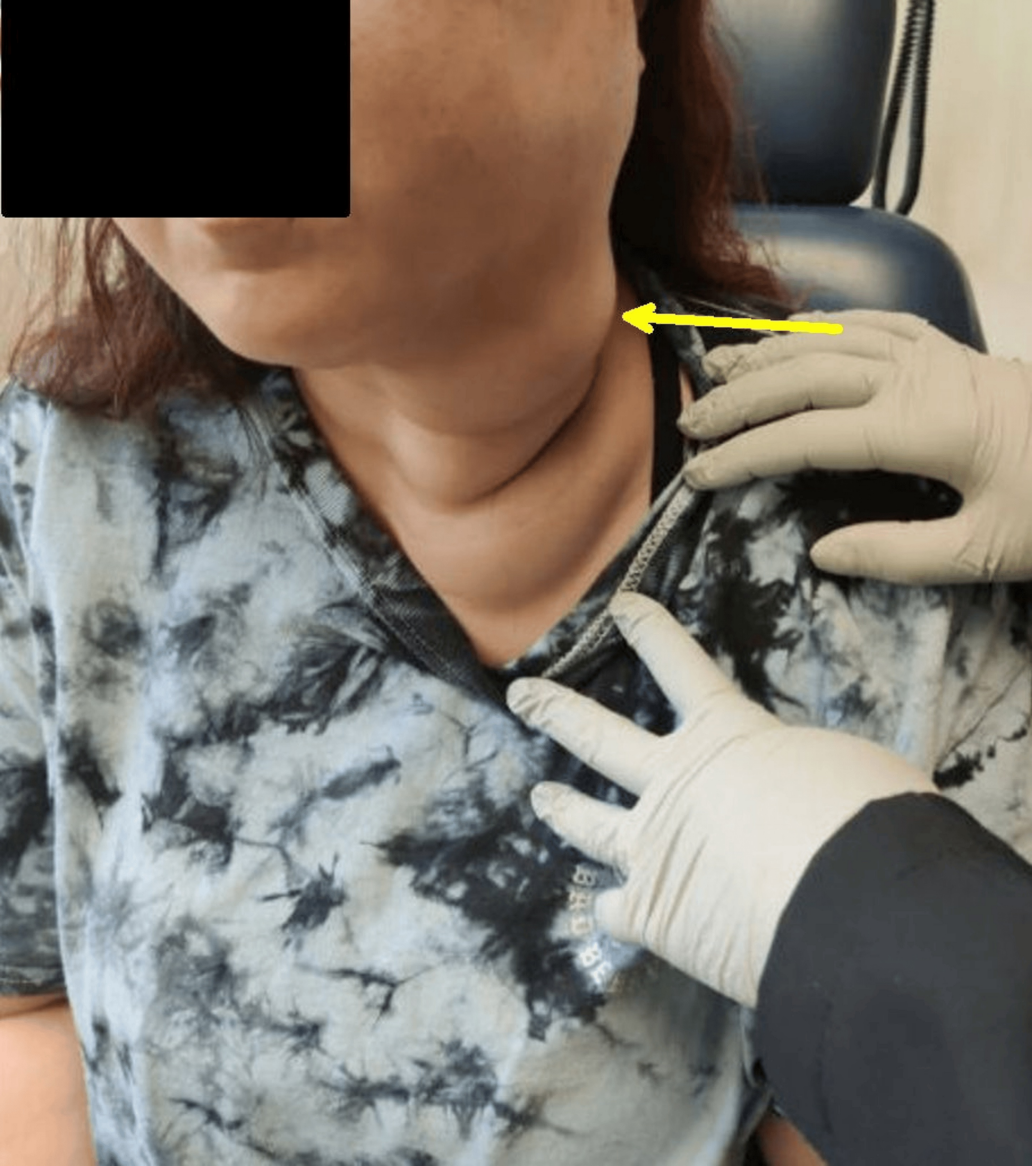
Clinical examination of neck region. Prominent superficial vasculature over the left neck and supraclavicular area (arrow).

On arrival, her vital signs showed tachycardia and mild hypotension. Hemoglobin was 8.1 g/dL (normal range 12.3 to 15.3 g/dL), WBC 11.2 × 10⁹/L (normal range 4.5 to 11.2 × 10⁹/L), and platelets 220 × 10⁹/L (normal range 100 to 400 × 10⁹/L). Physical examination revealed a firm, pulsatile mass extending from the left submandibular region to the tongue base, with mucosal discoloration and intermittent active oozing, signifying a high-risk lesion with direct airway and vascular compromise (Figure 2). TXA gauze packing was applied, and she was transferred for subspecialty evaluation.

**Figure 3:**
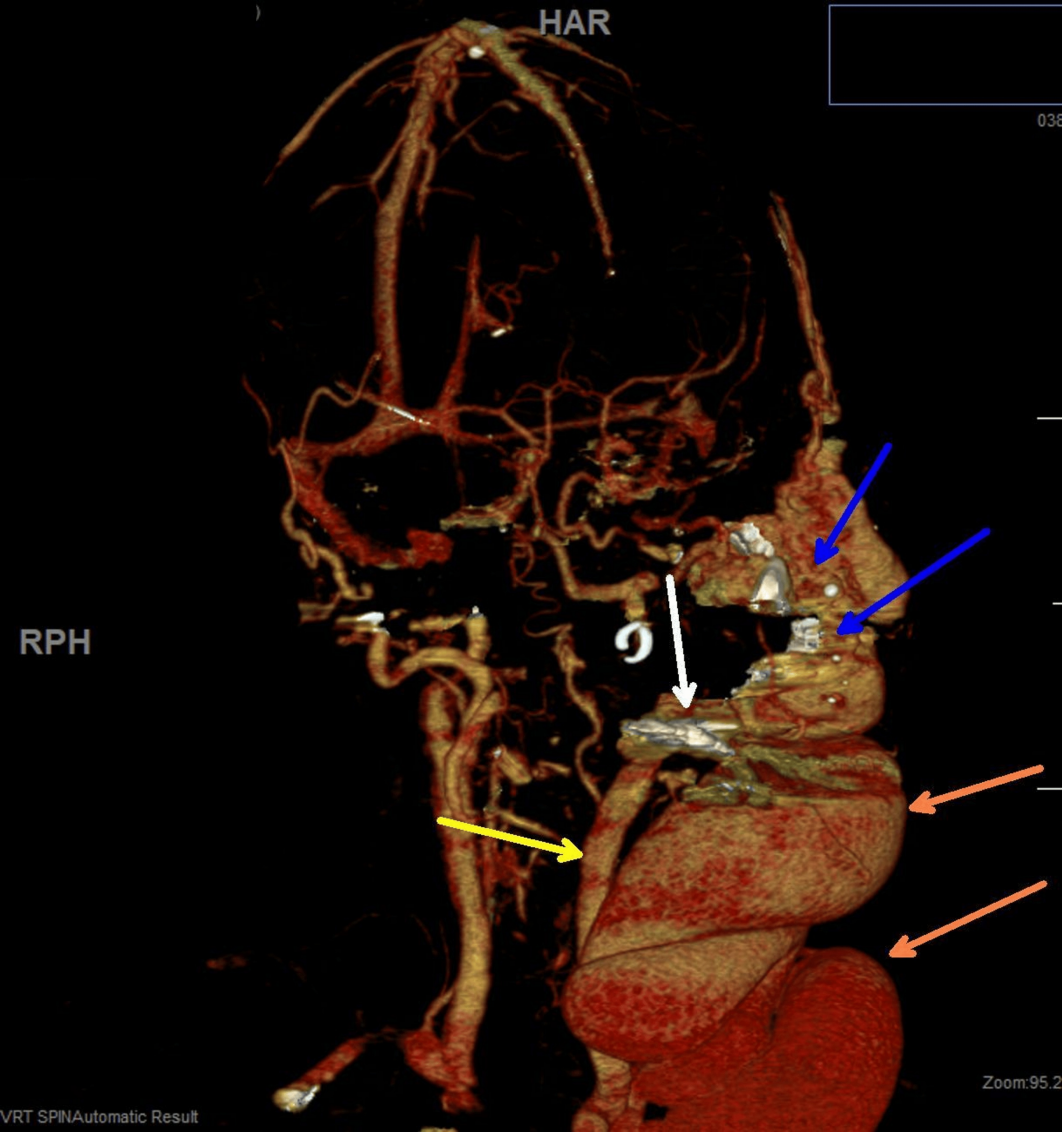
A slightly rotated MR angiogram of the head and neck to emphasize the soft tissue mass. The yellow arrow is the common carotid artery with a block at the bifurcation, with the white arrow showing residual blocked contrast in the external carotid artery after embolization because of no flow. The circular white arrow shows residual flow in the internal carotid artery in the head to the anterior cerebral artery and middle cerebral artery. The serpiginous AVM and the large draining left external jugular varix are at the orange arrow. The blue arrow indicates that the main arterial feeder from the left external carotid artery has been embolized, as well as embolization of right external carotid artery maxillary branch.

On hospital day 2, an MR angiogram of the head and neck revealed widespread involvement of the lingual and facial arterial systems. Onyx embolization resulted in partial flow reduction but was complicated by severe oral bleeding, requiring emergency airway management and intubation. The complex AVM appeared centered in the left parotid gland, extending to the left masticator space and extending inferiorly. The main arterial feeder from the left external carotid artery was embolized. As well as embolization of the right external carotid artery maxillary branch (Figure 3).

After the procedure, she experienced hemorrhagic shock, which necessitated a massive transfusion, TXA infusion, and brief vasopressor support.

Due to ongoing oropharyngeal bleeding and nutritional compromise, an open gastrostomy tube was placed on hospital day 5. Despite starting vincristine chemotherapy on hospital day 6, bleeding persisted, resulting in recurrent ICU admissions and repeated TXA-soaked gauze packing. Palliative care was consulted on hospital day 8 to manage symptoms, address anxiety, and support the family. Through palliative-led discussions, her goals of care were clarified, centering on her wishes for potential extubation, oral intake, and maximizing meaningful time with her young son. The palliative team coordinated multidisciplinary meetings with critical care, oncology, and surgical teams to align treatments with her values. Over the following weeks, recurrent hemorrhages and increasing transfusion needs indicated progressive AVM infiltration. Surgical and endovascular options were considered futile. The patient remained alert and actively participated in decision-making, expressing a desire for ongoing treatment that balanced comfort and family presence. After a massive hemorrhage and cardiac arrest on hospital day 25, she was resuscitated but remained critically unstable. Following renewed discussions with her partner, family, and the palliative team, the decision was made to prioritize comfort measures. Reasons for further curative attempts were deemed futile based on cumulative transfusion burden, recurrent shock, and anatomical inoperability. On hospital day 36, after planned compassionate extubation and organ donation after circulatory death (DCD), the patient expired peacefully, surrounded by her family.

## Discussion

This case is important for two main reasons: the aggressive clinical progression of a diffuse cervical AVM and the integration of palliative care within the ICU. Cervical AVMs are rare but highly severe vascular anomalies due to their proximity to major blood vessels and airway structures [7]. Diffuse AVMs, in particular, are defined by extensive arterial feeders, collateral vessel growth, and infiltration across multiple tissue planes, making them surgically unresectable and increasing the risk of recurrent or catastrophic bleeding [8]. Despite significant advances in endovascular embolization techniques and targeted medical therapies, outcomes remain poor once the lesion becomes diffuse and infiltrative [9]. Additionally, hormonal factors during reproductive years may worsen disease progression [10].

The second key aspect of this case is the significance of palliative care in the ICU, a setting traditionally focused on curative and life-sustaining treatments [11]. For patients with end-stage vascular conditions, palliative care provides a structured and ethically appropriate approach to symptom management, complex communication, and aligning medical interventions with the patient’s values and care goals [12]. A PubMed literature review revealed no prior studies specifically addressing the integration of palliative care for patients with lethal cervical AVMs in the ICU setting. This case report, therefore, addresses a significant gap, providing a clinical framework for this specific intersection of complex disease management and palliative principles. 

Existing evidence demonstrates that early integration of palliative principles in critical care enhances symptom management, increases family satisfaction, and can reduce unnecessary interventions and length of stay without compromising survival [13]. In this case, early palliative consultation facilitated clear goal-setting, offered psychological and emotional support, and supported a respectful transition to end-of-life care, illustrating the vital collaboration between critical and palliative care teams in managing complex, life-limiting illnesses.

## Conclusions

This case highlights the complex challenge of managing a severe cervical AVM while integrating palliative care principles within the ICU. For patients with advanced vascular anomalies unresponsive to curative treatments, early and collaborative involvement of palliative care teams is vital. Working with ICU staff, palliative care specialists help ensure patient-centered care, provide essential support to families, and facilitate compassionate transitions of care. This partnership is crucial for delivering comprehensive, respectful, and dignified care, particularly for young patients with rare, catastrophic illnesses where curative and palliative goals must be pursued concurrently from an early stage.

## References

[1] Shabbir F, Rashid M, Khan MI, Sarwar SU, Khan AH, Goher M (2024). Our experience in the surgical management of arterio-venous malformations of the head and neck. JPRAS Open.

[2] Kumar A, Mittal M, Srivastava D, Jaetli V, Chaudhary S (2017). Arteriovenous malformation of face. Contemp Clin Dent.

[3] Tanoue S, Tanaka N, Koganemaru M, Kuhara A, Kugiyama T, Sawano M, Abe T (2023). Head and neck arteriovenous malformations: clinical manifestations and endovascular treatments. Interv Radiol (Higashimatsuyama).

[4] Raman A, Uprety M, Calero MJ (2022). A systematic review comparing digital subtraction angiogram with magnetic resonance angiogram studies in demonstrating the angioarchitecture of cerebral arteriovenous malformations. Cureus.

[5] Erdmann MW, Jackson JE, Davies DM, Allison DJ (1995). Multidisciplinary approach to the management of head and neck arteriovenous malformations. Ann R Coll Surg Engl.

[6] Kozhevnikov D, Morrison LJ, Ellman MS (2018). Simulation training in palliative care: state of the art and future directions. Adv Med Educ Pract.

[7] Mulligan PR, Prajapati HJ, Martin LG, Patel TH (2014). Vascular anomalies: classification, imaging characteristics and implications for interventional radiology treatment approaches. Br J Radiol.

[8] Schimmel K, Ali MK, Tan SY (2021). Arteriovenous malformations-current understanding of the pathogenesis with implications for treatment. Int J Mol Sci.

[9] Pinkiewicz M, Pinkiewicz M, Walecki J, Zawadzki M (2022). State of the art in the role of endovascular embolization in the management of brain arteriovenous malformations—a systematic review. J Clin Med.

[10] Utami AM, Halfwerk JB, de Boer OJ (2023). Relative expression of hormone receptors by endothelial and smooth muscle cells in proliferative and non-proliferative areas of congenital arteriovenous malformations. Eur J Med Res.

[11] Christensen M, Liang M (2023). Critical care: a concept analysis. Int J Nurs Sci.

[12] Engel M, Kars MC, Teunissen SC, van der Heide A (2023). Effective communication in palliative care from the perspectives of patients and relatives: a systematic review. Palliat Support Care.

[13] Neukirchen M, Metaxa V, Schaefer MS (2023). Palliative care in intensive care. Intensive Care Med.

